# Reduction in intake of discretionary foods and drinks among Danish schoolchildren: dietary results from the real-life cluster-randomised controlled trial ‘Are You Too Sweet?’

**DOI:** 10.1017/S1368980024000740

**Published:** 2024-03-26

**Authors:** Sidse Marie Sidenius Bestle, Anne Dahl Lassen, Anja Pia Biltoft-Jensen, Jeppe Matthiessen, Sarah Jegsmark Gibbons, Bodil Just Christensen, Bjarne Kjær Ersbøll, Ellen Trolle

**Affiliations:** 1 Division of Food Technology, National Food Institute, Technical University of Denmark, Henrik Dams Allé, Building 202, Lyngby, Denmark; 2 Department of Applied Mathematics and Computer Science, Technical University of Denmark, Lyngby, Denmark; 3 Department of Biomedical Sciences, University of Copenhagen, Copenhagen, Denmark

**Keywords:** Child nutrition, Family-based intervention, Discretionary foods, School health nurse, Dietary guidelines

## Abstract

**Objective:**

To evaluate the effectiveness of the multicomponent intervention trial ‘Are You Too Sweet?’ in reducing discretionary foods and drinks intake among young schoolchildren.

**Design:**

The study was a 3·5-month two-arm cluster-randomised controlled trial among primary schoolchildren and their families. School health nurses provided guidance to families regarding discretionary foods and drinks for the children. Moreover, families were given a variety of knowledge- and capability-building materials to utilise at home. Dietary intake was assessed using a web-based 7-d dietary record. Linear mixed regression models were used to estimate intervention effects as changes in child intake of discretionary foods and drinks and sugar between groups.

**Setting:**

Six schools from a Danish municipality were randomised to the intervention group (*n* 4) or the control group (*n* 2).

**Participants:**

A total of 153 children aged 5–7 years.

**Results:**

No significant reduction in the children’s intake of total discretionary foods and drinks or discretionary foods alone was observed between the intervention and control group, while a decreased intake of discretionary drinks of 40·9 % (*P* = 0·045) was observed compared with control. Secondary subgroup analysis showed that children of parents with shorter educational level significantly reduced their intake of added sugar by 2·9 E% (*P* = 0·002).

**Conclusion:**

The results of this study indicate that multicomponent interventions involving school health nurses may have some effects in reducing, especially, discretionary drinks.

Developing implementable public health strategies focusing on healthy dietary habits early in life is of global importance^(1)^. A high intake of energy-dense and nutrient-poor food compromises the intake of important nutrients and core foods crucial for childhood development^(2–5)^. Furthermore, a high intake of added sugar, especially from sugar-sweetened beverages, is associated with the development of overweight, insulin resistance and poor dental health in children^(6–10)^.

Dietary surveys from several Western countries reveal that children get a large amount of their daily energy intake from energy-dense, nutrient-poor food and drinks^(11–14)^. How this food group is depicted in the literature varies from ‘extra foods’, ‘empty calories’ or discretionary foods but is all characterised by being rich in added sugar, solid fats and salt^(15)^. Data from the USA reports that 99·9 per cent of children aged 4–8 years exceed the recommended energy allowance from added sugar and solid fats, according to US federal dietary guidelines^(11)^. Australian children and adolescents (2–18 years) have been reported to receive almost 40 % of their daily energy intake from discretionary foods, such as sugar-rich food and drinks, takeaway foods, and processed meat^(12)^. The Dutch National Food Consumption Survey reports that 18–21 % of energy consumption (E%) in children and adolescents (7–18 years) comes from free sugars (added sugar, including sugars from fruit juice, honey, and syrups), of which over 80 % comes from sweets, candy, cakes and sugar-sweetened beverages^(13)^. In Denmark, Danish dietary survey data conclude that Danish children (4–6 years), on average, have an intake of 125 g/week of candy and chocolate, 385 g/week of cakes, ice creams, and energy-dense snacks^(14)^. Half of the Danish children exceed the recommendations from the WHO and Nordic Nutrition Recommendations of a maximum of 10 E% added sugar^(16–18)^. A qualitative study from Denmark suggests that although there is a general awareness of healthy and unhealthy foods, parents are not fully aware of how much is too much of discretionary choices^(19)^.

The Food and Nutrition Authorities in both Australia and the USA have made guidelines on discretionary intakes. The Australian guidelines, which include fast foods in the definition, allow for 0–½ serves of discretionary choices for children daily^(20)^. The Dietary Guidelines Advisory Committee in the USA has estimated a requirement of 87–94 E% essential calories for energy requirements below 3000 kcal/d, corresponding to a maximum of 6–13 E% from added sugar and solid fats^(21)^. Denmark has chosen to follow these examples, and based on an average Danish diet, the new maximum recommended intake of discretionary foods and drinks has been calculated by the Technical University of Denmark and defined as 4–6 % of total energy consumption, where discretionary foods and drinks include chocolate, candy, salty snacks, sugar-sweetened beverages, cakes and desserts^(15)^. These new guidelines have been communicated as limits of weekly servings and small servings for children and are now part of Danish official dietary guidelines^(22)^. Small servings, hereafter referred to as servings, are defined as approximately 450 kJ of solid discretionary foods or 250 ml of drinks. The maximum recommended weekly servings has been set to four for children 4–6 years of age and five for children 7–9 years of age^(15)^.

Following the development of the new guidelines, the intervention ‘Are You Too Sweet?’ was designed using a multicomponent theory-based approach involving school health nurses and families to limit the intake of discretionary foods and drinks among young schoolchildren. The Social Cognitive Theory was chosen as a guide for developing the intervention using several components, as it aims to describe cognitive determinants for behavioural changes^(23)^.

Parents are responsible for the home setting and structure of discretionary choices, and they act as role models for their children; hence, parental involvement in dietary intervention with children is essential^(24,25)^. Outcomes of previous studies have proven that a high degree of parental involvement in interventions with a focus on discretionary food and/or drink consumption among children has fostered greater reductions in consumption than if parents were not involved in the process^(24,25)^. One way to reach direct parental involvement is by collaborating with local health authorities in direct contact with parents, for example, school health nurses. A Swedish study has evaluated the effectiveness of collaboration with local school health nurses using existing health services for families to prevent childhood overweight, including improving dietary habits and physical activity with positive effects^(26,27)^. Further, as discretionary foods and drinks often increase through school age^(14)^, early intervention is important.

To our knowledge, studies evaluating specific guidance of maximum servings of discretionary foods and drinks in children are limited and more evidence-based strategies to reduce the intake of discretionary foods and drinks have been called for^(25,28)^. Furthermore, using existing healthcare services has the potential to ensure implementation and sustainable practices.

The aim of the current study was to evaluate the effectiveness of the intervention ‘Are You Too Sweet?’ in reducing the intake of discretionary foods and drinks among children starting school.

## Methods

### Study design and population

A detailed description of the methodology and theoretical background of the trial has been published^(29)^. Qualitative analyses and evaluation of the intervention and materials are also published^(30,31)^. The intervention ‘Are You Too Sweet?’ was conducted as a two-arm, parallel, cluster-randomised controlled trial in a Danish municipality (Hvidovre). Collaboration with the municipality facilitated that the trial involved six schools out of nine public schools in the municipality.

The trial involved young school starters (5–7 years) and their parents. During the spring of 2020, parents of children starting in one of the six schools in the summer of 2020 received a call from the local dental clinician with an invitation to participate in the trial and to obtain parental consent to be contacted by the research team before school health nurse consultation. Inclusion criteria were that the child started at one of the six schools and that at least one parent spoke Danish to complete dietary registration and questionnaire. Parents had received information about the trial in their digital post-box (e-boks) beforehand. After the school summer holiday, parents who accepted the initial invitation were called by a research staff member to confirm participation and schedule a meeting for the introduction and baseline questionnaire before the consultation with the school health nurse. Baseline meetings and enrolment were taking place continuously over 6 weeks prior to school health nurse consultations. Families were followed up approximately 3·5 months after the consultation. The entire intervention ran from October 2020 to March 2021.

The six schools were randomised to either an intervention or a control group. Power calculations prior to the trial were based on 6- and 7-year-old children in the Danish National Survey of Diet and Physical Activity^(16)^. For an 80 % power and a 95 % CI, calculations determined that seventy-six participants were required in each group to detect a 25 % reduction in sugar-rich discretionary foods by weight. For a 25 % reduction in added sugar, sixty-three participants were required. Practicalities and collaboration with the school health nurses required randomisation to occur before the baseline measures, but participants were blinded for randomisation until after the completion of baseline measures. Randomisation was conducted by the research team, and R statistical software was used for randomisation to ensure an even distribution of the schools’ socio-economic index and number of children. A larger intervention group was used to ensure statistical power, as there was an early large dropout partly due to the COVID-19 pandemic after the summer holiday before baseline appointments were made. Thus, four schools were randomised to the intervention group and two schools were randomised to the control group^(29)^.

### Intervention components

In Denmark, all families are provided a consultation with the school health nurses within their child’s first year of school. The intervention ‘Are You Too Sweet?’ was integrated into this existing practice. The intervention was designed to provide updated guidelines on discretionary foods and drinks, specifying the maximum number of servings to be consumed weekly. A maximum of four weekly servings was recommended for children from 4 to 6 years of age. Of those, one of the servings can be a discretionary drink of 250 ml^(15)^. The intervention consisted of three parts: (1) an extended consultation with the school health nurse, from 30 to 35 min, that is, 5 min focusing on discretionary foods and drinks. This included an evaluation of answers from a validated fast digital sugar-rich food screener^(32)^ that parents filled out before the consultation, aiming to qualify the dialogue about their child’s ‘sweet’ habits. Both the parents and school health nurses received the resulting output. (2) A use-at-home box containing intervention materials: an inspiration booklet with recommendations, an educational card game, a serving-size board with stickers, stickers for an AR app game, a children’s book, tickets for the local swimming pool and local activity suggestions. The school health nurses handed out these boxes. The materials were selected and designed to offer concrete advice, illustrate recommendations and provide inspiration to follow recommendations and engage in activities. (3) An invitation to join a private Facebook group to provide prompting posts and support interaction among participating parents.

Common to both the intervention group and the control group was that all school health nurses received up-to-date guidelines on diet, physical activity, screen time and sleeping patterns to use for all school consultations. This provided uniform guidance principles for consultations from school health nurses across different schools.

### Procedure and outcome measures

Parents of participating children were invited to an introduction to the project at baseline. During the introduction, they were asked to complete a questionnaire about educational, parental practices, self-efficacy, sleep and dental health. Parental educational level was summarised for both parents, and the parent with the longest educational level was reported. Long educational level was thus defined meaning at least one parent with at least 14 years of education (bachelor’s degree or longer), while shorter educational level was defined as neither of the parents had a bachelor’s degree. The questionnaire was tested by a think-aloud test among six parents of children between 5 and 9 years of age and by a feasibility test with nineteen parents beforehand. Parental practices and self-efficacy are not reported in this paper. Parents were also instructed to fill out a self-administered web-based 7-d dietary record on behalf of their child, starting the day after the introduction. The dietary assessment software for this purpose was structured by six eating occasions a day: breakfast, lunch, and dinner, and three in-between meals after breakfast, lunch, and dinner. For each meal, parents had to search for food items from a food list of 1,710 items and choose an appropriate portion size from one to four pictures or fill out an open-answer option if the food item could not be found. At the end of the registration, parents were asked if they forgot to register their child’s intake of sweets or chocolate, if their child had any nutritional supplements and if the day represented a usual or unusual day with reasons such as a birthday or illness. A similar web-based dietary assessment tool has previously been validated among children^(33)^. If parents forgot to register for a day, they were reminded by email the following day and received a text message after 2 d of missing registrations. Two to 3 weeks after the introduction, parents and their child were invited to a consultation with the school health nurse and were informed whether their child belonged to the intervention or control group. The school health nurse measured the child’s height and weight for all children which is part of the standard consultation and handed out the use-at-home box with intervention materials to the children in the intervention group.

After approximately 3·5 months of intervention, parents from both the intervention and control groups were asked to fill out the 7-d dietary record again. Parents and their children were invited to a follow-up consultation with the school health nurse, where they answered a follow-up questionnaire, and the child’s height, weight, and waist circumference were remeasured. Following the intervention, two focus groups were conducted with all twelve school health nurses involved in the ‘Are You Too Sweet’ project, and evaluation interviews were conducted with twenty-four intervention families, with results published elsewhere^(30,31)^.

The primary outcomes in the current study were child intake of discretionary foods and drinks, as defined in the recommendations, measured as servings and energy summarised and separately analysed. Secondary outcomes were changes in the intake of subgroups of discretionary foods and drinks reported in servings. One of the messages delivered during the intervention was to substitute discretionary foods and drinks with healthier choices, such as fruits or wholegrain crispbread. An analysis of vegetables, fruits and wholegrain products was also conducted. Finally, changes in the overall dietary composition of macronutrients and total energy intake were analysed, including intake of added sugar. As a large amount of children’s intake of added sugar comes from discretionary choices, reduction in added sugar was an important part of improving children’s dietary quality.

### Food intake estimates and statistical considerations

Intake of food items, energy and nutrients were calculated for each child for each meal and as an average intake per d using the software system General Intake Estimation System (GIES) version 1.000.i6 and the Danish Food Composition Databank version 7.0, both developed at the National Food Institute, Technical University of Denmark. At least 4 d, including at least one weekend day and three weekdays, were required for a valid dietary recording. The average daily intake of food items and nutrients was aggregated, and an average per d consumption was estimated for each participant. Intake data were aggregated using the Tidyverse package in R software version 4.0. Over- and under-reporters of dietary intake were identified by evaluating reported energy intake using the Goldberg cut-offs for the ratio between reported energy intake and estimated BMR at the individual level, as recommended by Black^(34)^. BMR was calculated using Henry’s sex-specific equations^(35)^ with the use of weight and height measured by school health nurses and the physical activity level set to 1·57, as proposed by The European Food Safety Authority (EFSA)^(36)^. All further analyses were adjusted for reporting status.

Intake of discretionary foods and drinks were aggregated for each participant and assessed as energy intake and servings. In this intervention, servings constituted the units communicated in the recommendations and are thus proxies equalising food and drinks across different energy densities, including artificially sweetened drinks that were also considered discretionary. The procedure defining and categorising discretionary foods and drinks were done by nutrient profiling from a food item list, where high energy density and low nutrient density foods were categorised as discretionary. Food items from the food list used in the dietary record that were considered discretionary cover foods such as chocolate, candy, cakes, pastries, desserts, ice cream, biscuits, salty snacks, crackers, and sugar-sweetened and artificially sweetened drinks. Nutrient information was retrieved from the Frida database, version 4^(37)^. The process has been thoroughly described elsewhere in the context of the new guidelines^(15)^.

Changes in the intake of discretionary foods and drinks in means of energy and servings per d and changes in total energy intake and macronutrients were analysed using linear mixed models as repeated measurements, including participant and school as random effect. The interaction effect between time measurement and group was reported as the effect size. If food groups or drinks contained data with zero intakes, they were given an intake corresponding to the lowest value detected divided by two to log-transform a non-normally distributed (right-skewed) intake. All models were adjusted for school and child as random effects, and parental education, sex, BMI, and misreporting as fixed effects. Sensitivity analyses were performed in two ways: first, for primary outcomes by models, not adjusting for other covariates, only adjusting for random effects, and second, complete cases analysis by comparing follow-up measures between groups, adjusting for baseline values (ANCOVA).

In a *post hoc* analysis, changes in intake during weekdays and weekend days were analysed separately as a significant variation in discretionary foods and drinks intake between weekdays and weekends has been found among Danish children^(38)^. Friday was regarded as a weekend day, as a large part of the intake of discretionary foods and drinks is consumed on Fridays as a part of family custom for ‘Friday sweets’ in Denmark^(31,38)^.

## Results

**Fig. 1:**
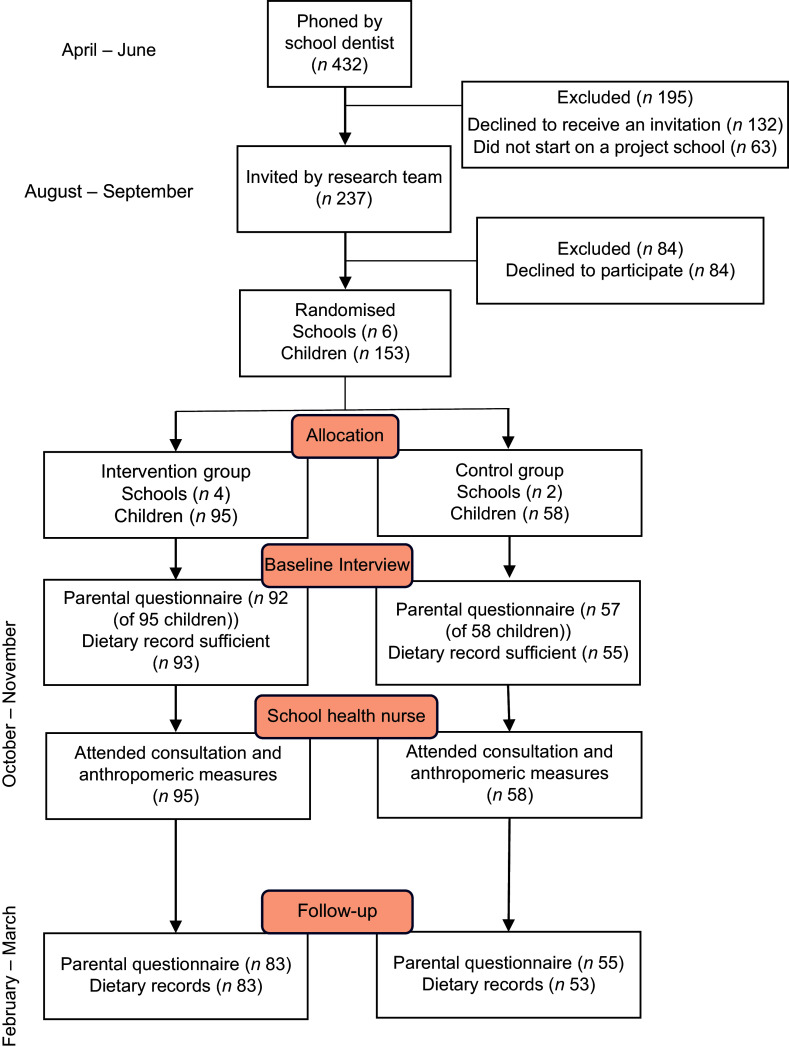
Flow diagram of intervention and participants in ‘Are You Too Sweet’

Two hundred and thirty-seven families with children planned to start at one of the six project schools were initially contacted during the spring before the start of school. After the summer holiday, 153 children and their parents were enrolled in the intervention, ninety-four in the intervention group and fifty-six in the control group. The most common reason for declining participation was a lack of time and resources. Less than half of the children from each school participated in the study. Dietary records from five children were considered insufficient at baseline, while 148 completed the dietary record according to the protocol. At follow-up, thirteen children dropped out, and dietary records from four children were considered insufficient. Dietary data were valid for 136 children at follow-up, corresponding to 89 % of the children at baseline. In the final analysis, 150 children participated with data. A flow diagram of the participants is provided in Fig. 1.

The baseline characteristics of the 150 children used in the analysis are shown in Table 1. Most children were 6 years of age at their first consultation with the school health nurse. Parental educational level differed between the intervention and the control group, which can be explained by the fact that randomisation occurred at school level in areas with different socio-economic statuses. Children were evenly distributed by sex, with 52 % and 48 % girls in the intervention and control groups, respectively. Most children were regarded as normal weight by International Obesity Taskforce (IOTF) standards^(39)^. More children, but not significantly, were categorised as overweight in the intervention group compared with the control group (14 % and 9 %, respectively) (Table 1).

**Table 1:** Descriptive characteristics of enrolled children and their parents

	Intervention group (*n* 94)		Control group (*n* 56)	
	Mean	sd	Mean	sd
*Child* Age	6·4	0·3	6·4	0·3
BMI	15·7	1·7	15·5	1·2

	%		%	
Sex; female	52		48	
Weight status, IOTF Overweight or obese	14		9	
*Parents* Education level (longest of at least one parents) >14 years, bachelor’s degree	52		71	
Ethnicity (maternal), non-Western	7		5	

IOTF, International Obesity Taskforce.

At baseline, the children had a median intake of 2·2 and 2·5 daily servings of discretionary foods and drinks in the intervention and control groups, respectively, and 1·9 and 2·1 servings daily at follow-up (Table 2). This corresponds to 15·4 and 17·5 weekly servings of discretionary foods and drinks in the intervention and control groups, respectively, at baseline. The difference over time in discretionary foods and drinks, both in means of energy and servings, was observed to be significantly lower (14·1 % and 14·7 %, respectively) at follow-up for the intervention group. Further, a significant lower intake of discretionary drinks was observed within the intervention group from baseline to follow-up (Table 2).

**Table 2:** Child intake of selected food groups in the intervention and control groups, at baseline and follow-up, respectively

Food groups	*n*_*0*_ *	Intervention group						*n*_*0*_ *	Control group					
		Baseline (*n* 93)		Follow-up (*n* 83)		Change†			Baseline (*n* 55)		Follow-up (*n* 53)		Change†	
		Median	IQR	Median	IQR	Est (%)	95 % CI		Median	IQR	Median	IQR	Est (%)	95 % CI
Discretionary foods and drinks (kJ/d)	0/0	930	602; 1347	821	553; 1063	−14·1	**−25, –1·7**	0/0	1102	702; 1357	860	684; 1173	−1·8	−13·7, 11·8
Discretionary foods and drinks (s/d)	0/0	2·2	1·4; 3·1	1·9	1·3; 2·6	−14·7	**−25·8, –1·9**	0/0	2·5	1·6; 3·3	2·1	1·7; 2·8	0·8	−11·4, 14·7
Discretionary drinks (ml/d)	20/28	57	14; 147	43	0; 90	−37·0	**−52·8, –15·9**	11/11	50	14; 89	47·1	17·9; 114·3	8·2	−26·9, 60
Discretionary drinks (s/d)	20/28	0·2	0·1; 0·6	0·2	0; 0·4	−39·5	**−55·8, –17·2**	11/11	0·2	0·1; 0·4	0·2	0·1; 0·5	8·7	−29·3, 67·2
Discretionary solid food (s/d)	0/1	1·9	1·1; 2·6	1·7	1·1; 2·2	−10·4	−22·4, 3·4	0/0	2·2	1·5; 2·8	1·8	1·2; 2·5	−4·6	−16·1, 8·6
Candy, chocolate (s/d)	2/5	0·4	0·3; 0·7	0·4	0·2; 0·6	−19·8	−38·1, 3·8	2/1	0·6	0·4; 1	0·5	0·3; 0·8	−11·5	−34·2, 19·1
Desserts, cakes, ice cream (s/d)	30/41	0·6	0·3; 1·1	0·5	0·2; 1	−19·2	−44·7, 17·9	23/27	0·7	0·5; 1·2	0·7	0·3; 1·3	−22·8	−54·7, 31·7
Crackers, bars (s/d)	24/25	0·3	0; 0·5	0·1	0; 0·4	−23·4	−45, 6·7	11/14	0·3	0·1; 0·6	0·2	0; 0·4	−17·7	−49·8, 34·8
Salty snacks (s/d)	32/23	0·2	0; 0·4	0·2	0; 0·5	27·2	−15·5, 91·7	20/20	0·1	0; 0·5	0·1	0; 0·5	−3·7	−40·3, 55·4
Fruits and vegetables (g/d)	0/0	223	166; 315	241	149; 311	−7·7	−15·6, 0·9	0/0	242	153; 306	222	147; 268	−4·4	−17·8, 11·2
Wholegrain products (g/d)	0/0	76	54; 97	75	48; 103	−9·6	−23·3, 6·5	0/0	82	54; 106	68	42; 91	−23·9	**−36·6, –8·8**

s/d, servings per d (servings defined as serving sizes of 450 kJ or 250 ml discretionary drinks); IQR, interquartile range; Est, estimate.

* Numbers of non-consumers, baseline/follow-up, are showing cases of zero intakes. Values of half the minimum value of the sample were imputed to ensure model validation and make log-transformation possible.

† Log-transformed mixed models estimating mean difference from baseline to follow-up. Estimates are given in percentage as models are log-transformed. Adjusted for parental education, misreporting, sex and BMI as fixed effects, and school and participant as random effect. *P*-values <0·05 are bolded.

In both the intervention and control groups, a significant decrease energy intake was observed at follow-up compared with baseline (–299 kJ/d and –268 kJ/d, respectively) (Table 3). Intake of added sugar was significantly lower at follow-up compared with baseline in the intervention group (–1·6 E%) but not in the control group. Further, subgroup analysis on educational level revealed a difference for children of parents with lower educational level in the intervention group but not the children of parents with higher parental educational level (Table 3). In the intervention and control groups, the proportion of mis-reporters (mainly under-reporting) increased from baseline to follow-up, from 8 % to 18 % and 10 % to 18 %, respectively (not shown in tables).

**Table 3:** Child intake of selected nutrient groups in the intervention and control groups, at baseline and follow-up, respectively

Variable	Intervention group						Control group					
	Baseline (*n* 93)		Follow-up (*n* 83)		Change†		Baseline (*n* 55)		Follow-up (*n 53*)		Change†	
	Mean	sd	Mean	sd	Est	95 % CI	Mean	sd	Mean	sd	Est	95 % CI
Total energy intake (kJ/d)	5868	1312	5430	1112	−299·3	**−549, –49·5**	5863	1128	5461	1240	−268	**−471, –64**
Total fat (E%)	34	4·5	34·7	4·7	0·9	−0·1, 1·8	33·8	3·4	34·7	4·2	1·2	−0·1, 2·4
Saturated fat (E%)	12·7	2	12·6	2·4	−0·1	−0·5, 0·4	12·2	2	12·5	1·9	0·3	−0·2, 0·9
Carbohydrates (E%)	49·9	4·7	48·2	4·8	−1·6	**−2·5, –0·7**	50·2	3·8	49·1	4·5	−1·3	**−2·5, –0·1**
Added sugar, all (E%)	8·1	4·3	6·2	3·4	−1·6	**−2·4, –0·9**	8·4	3·5	7·7	3·4	−0·7	−1·6, 0·3
Added sugar, upper parental education (E%)*	7·1	4·0	6·2	3·3	−0·8	−1·8, 0·3	8·7	3·7	7·5	3·5	−1·1	−2·2, 0·1
Added sugar, lower parental education (E%)*	9·2	4·3	6·2	3·4	**−2·8**	**−3·9, –1·8**	7·8	2·8	8·0	3·3	0·4	−1·1, 1·9
Protein (E%)	14·7	2·3	15·6	2·4	0·7	0·3, 1·2	14·7	1·7	14·9	1·7	0·2	−0·2, 0·7

E%, percentage of total energy intake; Est, estimate.

* As a significant interaction effect between group *X* measure time *X* parental education was found, subgroup analysis was performed.

† Mixed models estimating mean difference from baseline to follow-up. Adjusted for parental education and misreporting as fixed effects, and school and child as random effect. *P*-values <0·05 are bolded.

The relative difference in the intake of discretionary foods and drinks between the intervention and control groups at follow-up was not significant. However, the estimated decrease in the total servings of discretionary foods and drinks by 15·4 % tended to be borderline significant (*P* = 0·099), and a decreased intake in servings of discretionary drinks analysed separately was observed by 40·9 % (*P* = 0·045) (Table 4). No significant intervention effect was found for other food groups or nutrients. For added sugar, an interaction effect was found for parental educational level; subgroup analysis by parental educational level was performed and showed that children of parents with shorter educational level significantly reduced their intake of added sugar by 2·9 E% (*P* = 0·002). In contrast, no significant change was found for children of parents with high educational levels (Table 4).

**Table 4:** Child intake of selected nutrient groups in intervention and control group, at baseline and follow-up, respectively

Food or nutrient group	Between group change*		
	Estimate (%)†	95 % CI	*P*-value
Discretionary foods and drinks (kJ/d)	−12·3	−27·8, 6·6	0·187
Discretionary foods and drinks (s/d)	−15·4	−30·6, 3·2	0·099
Discretionary drinks (ml/d)	−38·6	−61·5, –1·9	**0·041**
Discretionary drinks (s/d)	−40·9	−64·6, –1·3	**0·045**
Discretionary solid foods (s/d)	−5·8	−23·1, 15·4	0·564
Candy and chocolate (s/d)	−5·3	−36·3, 40·9	0·790
Desserts, cakes and ice cream (s/d)	8·2	−42, 102·1	0·804
Crackers and bars (s/d)	−3·5	−45, 69·3	0·901
Salty snacks (s/d)	28·4	−31·5, 140·5	0·436
Fruits and vegetables (g/d)	−3·3	−17·6, 13·5	0·685
Wholegrain products (g/d)	17·1	−8·6, 50	0·211

	Estimate (mean)	95 % CI	*P*-value
Total energy intake (kJ/d)	−2·9	−350·3, 344·5	0·987
Total fat, E%	−0·2	−1·7, 1·3	0·793
Saturated fat, E%	−0·4	−1·1, 0·3	0·215
Carbohydrates, E%	−0·4	−1·9, 1	0·547
Added sugar, all E%	−0·9	−2·1, 0·3	0·131
Added sugar, upper parental education, E%‡	0·2	−1·3, 1·8	0·757
Added sugar, lower parental education, E%‡	−2·9	−4·8, –1·1	**0·002**
Protein, E%	0·6	−0·1, 1·3	0·08

s/d, servings per d (servings defined as serving sizes of 450 kJ or 250 ml discretionary drinks); E%, percentage of total energy intake.

* Log-transformed mixed models estimating interaction effect time *X* group. Adjusted for parental education, misreporting, sex and BMI as fixed effects, and school and participant as random effect.

† Estimates are presented as percentage as the outcome has been log-transformed.

‡ As a significant interaction effect between group *X* measure time *X* parental education was found, subgroup analysis was performed. *P*-values <0·05 are bolded.

Sensitivity analysis with unadjusted models (model 1) and models using ANCOVA by complete cases to compare intervention and control groups at follow-up (Model 2) showed similar results with estimates in the same range, regarding changes in discretionary foods and drinks. However, although the change in discretionary drinks showed similar estimates, findings were non-significant in the ANCOVA analysis (model 2) (see online supplementary material, Table S1). *Post hoc* analysis stratifying by weekdays (Monday–Thursday) and weekend days (Friday–Sunday) showed a significant decrease in the mean intake of servings per d of discretionary foods and drinks on weekend days in the intervention group compared with the control group by 39 % (*P* = 0·003) (see online supplementary material, Table S2).

## Discussion

This study analysed the effectiveness of the multicomponent intervention trial ‘Are You Too Sweet?’ The intervention aimed to reduce the intake of discretionary foods and drinks among young schoolchildren by providing new guidelines delivered through local school health nurses and knowledge- and capability-building materials. No significant intervention effects were found for children’s intake of discretionary foods and drinks summarised or in discretionary solid foods alone, while a significant decrease in the intake of discretionary drinks alone was observed. However, the intake of discretionary foods and drinks at baseline and follow-up differed significantly in the intervention group. Given the choice of a small control group, a decreased intake within the intervention group might suggest some influence of the intervention, as does the significant decrease in added sugar. Thus, a decreased intake of discretionary drinks might also be reflected in the summarised group as well as in added sugar intake. Although a significant decrease in discretionary drinks was found, the sensitivity analysis using complete cases could not confirm these findings, and the estimate was similar (model 2, see online supplementary material, Table S1). As many participants did not report any consumed discretionary drinks, these findings might lack robustness due to the large variation.

An Australian randomised controlled trial is focusing on discretionary foods in lunchboxes at school by promoting swapping strategies to 5–12-year-old children and their parents. They found a reduction of 117 kJ/d in lunchboxes compared with the comparison group^(40)^. However, the Australian trial had a narrower scope and a higher baseline intake among children. In the current study, the baseline intake was lower and might impact how large reductions can be observed. Large variation in intake of these foods and drinks is also worth noting. Thus, in the current study, subgroup analysis showed that added sugar intake was decreased in children of parents with lower educational levels but not for children of parents with higher educational levels. This finding could both reflect a social-influenced knowledge gap and help to reduce the added sugar intake especially in the children with the highest sugar intakes. Changes in added sugar were an interesting result because communication in the intervention focused on ‘sweet’ snacks, foods and drinks.

The intervention effect observed for discretionary drinks in the present study aligns with the previously published qualitative findings from the ‘Are You Too Sweet’ intervention, where parents reported especially cutting discretionary drinks, which reflects that awareness of the recommendation, especially on discretionary drinks, was successful in this intervention^(31)^.

In other studies, changes in snacking behaviour have likewise proven difficult. For instance, a large-scale European RCT, the ‘ToyBox-study’, delivered across six countries, focused on improving health regarding eating and snacking, physical activity, and sedentary behaviour among pre-schoolers by delivering tips and newsletters over 24 weeks, including educational advice concerning snacking to teachers and parents. With 4,970 children, they found no changes in snacking behaviour post-intervention^(41)^.

How strategies to reduce discretionary foods and drinks influence overall dietary patterns, for example, by substitution effects, is a relevant issue in future studies to optimise health promotion strategies. The current study detected a borderline increase in E% of protein but not in other food groups. Greater power is probably needed to make conclusions on this question. Further, a lower energy intake at follow-up might be caused by a fatigue effect of dietary registration, causing a biased estimate of changes.

The present study found that participating children consumed more discretionary foods and drinks on weekends than on weekdays, consistent with previous findings from Denmark and Australia^(38,42)^. *Post hoc* analysis found a significant reduction of discretionary foods and drinks on weekends, while no significant change was found during weekdays. This may seem to contradict the qualitative evaluation of the intervention ‘Are You Too Sweet?’ where parents reported reductions primarily on what they regarded as ‘everyday treats’^(31)^. However, ‘everyday treats’ were considered as treats not necessarily connected to weekdays but as a contrast to ‘socialised treats’ or ‘family treats’. Moreover, the families interviewed reported that giving smaller serving sizes^(31)^ could particularly affect the intake on weekend days. In some families, weekday intake was relatively low in the home setting, and some parents reported that they are not serving large amounts of discretionary treats^(31)^. Further, as consumption during weekend days is outside the school context, this highlights the importance of involving parents and the home setting in interventions focusing on discretionary foods and drinks.

In the qualitative evaluation of this trial, parents reported that it was difficult to make changes for intake outside the home in social settings^(31)^. A review by Johnson et al. on reducing discretionary food intake in children likewise highlights that intake of discretionary foods, including fast foods, was found to be associated with meals eaten away from home^(25)^. Future interventions might benefit from finding ways to target both the home and structural settings outside the home. In the ‘Are You Too Sweet’ intervention, not all children across a school class participated in the trial, and social and structural settings did not change. In this context, national, regional or even local differences in children’s intake of discretionary foods and drinks might be relevant when developing interventions.

An apparent strength of this study is that it was developed in collaboration with local school health nurses in a Danish context, aimed at implementation and upscaling. The intervention was designed as a real-life trial developed collaboratively with school health nurses who meet all children across social settings. Scaling up health promotion, developing implementable public health interventions and ensuring external validity have previously been discussed and highlighted as continuing issues in dietary or health-promoting interventions^(43,44)^.

A general limitation in studies using child dietary data is that the parent registration might be prone to recall bias as they are limited in observing their children’s intake outside of the home^(45)^. A strength, however, was that the child’s whole diet was measured through a 7-d dietary record, which allowed estimating energy intake from different food groups and calculating potential misreporting of energy. However, a limitation was that the proportion of mis-reporters, especially under-reporters, tended to increase at follow-up, indicating a potential fatigue effect of dietary registration and, thus, an untidier registration. This could be further reflected in a significant decrease in energy intake over time. Misreporting was adjusted, and bias would likely be evenly distributed between the intervention and control groups. The communication in the intervention was on ‘sweet’ snacks, foods and drinks with connotations of sugar content. Thus, although salty snacks such as crisps counted as discretionary, substitutions to those might have occurred. However, from the analyses, such substitutions are not found.

Due to the ‘real-life’ study design, the control group could not be blinded. Families in the control group were thus aware of the project’s overall focus and could be prone to selection bias as participation in this trial was voluntary. Furthermore, as the school health nurses across intervention and control schools received updated materials and were also engaged in the intervention, the control group can be described as a minimum intervention group more than a proper control group.

A main limitation of the study was the large drop in the number of participating families after the summer holiday, partly due to COVID-19. Power calculations prior to the intervention indicated a requirement of seventy-six participants in each group for a 25 % reduction in discretionary foods and drinks^(29)^. Thus, an underpowered control group was used to ensure power in the intervention group alone. The findings imply that the study might lack the power to detect significant differences, both by a low number of children and, perhaps even more importantly, with only two schools (clusters) in the control group. While power calculations prior to the study were conducted with an expectation of a 25 % decrease in intake, an effect estimates of a 12·3 % decreased energy of discretionary foods and drinks was also lower than expected. Thus, within-group changes observed might support that some effect might be found with more participants.

Another major limitation of the intervention was that a school lockdown started during the intervention period. A survey on habits during the COVID-19 lockdown showed that dietary patterns contain more discretionary choices^(46)^. Unpublished data from a questionnaire used in the intervention^(29)^ indicated the same pattern. Forty-six per cent of the parents in the intervention group reported that their child had increased their consumption when asked, ‘*How do you assess that the homeschooling after the Christmas holidays has affected [child’s name] intake of sweet treats and sweet drinks?’* Therefore, the intervention effects might have been greater at other times.

One of the overall aims of the ‘Are You Too Sweet?’ project was to halt the development of increasing the intake of discretionary foods and drinks throughout the school years. Although the effect was limited, the change could indicate an increased awareness among some families, supported by the qualitative evaluation that could influence long-term habits related to discretionary intake. The intervention may be improved by supplementing initiatives. Some parents expressed that the lack of school policies supporting the intervention hindered compliance and motivation, such as birthday celebrations or after-school activities. Implementation involving all children at the school might further limit social impediments for making dietary changes^(31)^. In line, the need for upstream intervention policies, for example, targeting school policies, marketing, exposure and availability of discretionary choices, has been underscored^(47,48)^.

### Conclusion

The results of this study indicate that the multicomponent intervention involving school health nurses may have some effects on reducing, especially, discretionary drinks. Although the results of the present study could not detect a significant decrease in the total amount of discretionary foods and drinks as an effect of the intervention ‘Are You Too Sweet?’, the analyses from this study suggest that providing individualised guidance, together with materials with a specific focus on discretionary foods and drinks, through school health nurses might have the potential to change the intake of these for some children, and especially regarding discretionary drinks and intake during weekends. Subgroup analysis on parental educational level showed that children of parents with lower educational levels reduced their intake of added sugar, likely due to the intervention’s focus on ‘sweet’ snacks, foods and drinks. Specific recommendations and individualised guidance that increase the awareness of discretionary foods and drinks might be essential tools and motivational factors for healthier dietary habits among children. Interventions that are scalable using existing or implementable healthcare services, like the present study, have the potential to define evidence-based practices in future public health promotion, preferably strengthened by supporting initiatives and policies. However, more studies are needed to confirm the effectiveness of the strategies, including analyses of which population groups might be more prone to benefit from such initiatives.

## Supporting information

Bestle et al. supplementary material Bestle et al. supplementary material

## Study registration

The study is prospectively registered at ISRCTN with the number: ISRCTN10409779.

## Supplementary material

For supplementary material accompanying this paper visit https://doi.org/10.1017/S1368980024000740
