# Supplementary material for: Reduction in intake of discretionary foods and drinks among Danish schoolchildren: dietary results from the real-life cluster-randomised controlled trial ‘Are You Too Sweet?’
Source: Public Health Nutr. 2024 Mar 26;27(1):e111. doi: 10.1017/S1368980024000740 (PMC11036427; doi:10.1017/S1368980024000740)
Supplement: Bestle et al. supplementary material [file S1368980024000740sup001.docx]

**Table S1. Sensitivity analysis of primary outcomes**

|  | **Model 1*** | | |  | **Model 2^†^** | | |
| --- | --- | --- | --- | --- | --- | --- | --- |
|  | **Estimate, %^††^** | **95% CI** | ***P*-value** |  | **Estimate, %^††^** | **95% CI** | ***P*-value** |
| Discretionary foods and drinks, kJ/d | -14.2 | -30.1;5.4 | 0.144 |  | -20.8 | -40.2;5 | 0.105 |
| Discretionary foods and drinks, s/d | -17.2 | -32.7;1.9 | 0.074 |  | -23.3 | -43.4;4 | 0.088 |
| Discretionary drinks, ml/d | -39.6 | -62;-4 | **0.033** |  | -42.1 | -70.8;15 | 0.119 |
| Discretionary drinks, s/d | -41.9 | -65;-3.6 | **0.035** |  | -45.2 | -74.4;17.3 | 0.121 |
| Discretionary solid foods, s/d | -7.9 | -25.9;14.4 | 0.457 |  | -17.2 | -36.3;7.7 | 0.159 |

* Model 1, is performed as a repeated measure analysis with estimates as the interaction of time and group, but unadjusted with only schools and id included as random effect.

† Model 2, is performed as a complete case analysis by ANCOVA, comparing intervention and control groups at follow up, adjusted for baseline intake, BMI, sex, misreporting, parental education as fixed effects, and school cluster as random effect.

†† Estimates are presented as percentages, as models have been log-transformed to fit a normal distribution.

**Table S2.** Changes in intake of discretionary foods and drinks during weekdays and weekend for intervention group, control group, and between groups after intervention period

|  | Intervention group  (Baseline, *n* 93), (Follow-up, *n* 83) | | | | | Control group  (Baseline, *n* 55), (Follow-up, *n* 53) | | | | |  |  |  | |
| --- | --- | --- | --- | --- | --- | --- | --- | --- | --- | --- | --- | --- | --- | --- |
|  |  |  |  |  |  |  |  |  |  |  | Between group change after intervention period | | |  |
|  | Baseline | | Follow-up | |  | Baseline | | Follow-up | |  | Change coefficient† | |  |  |
| Discretionary foods and drinks (s/d) during: | Median | IQR | Median | IQR |  | Median | IQR | Median | IQR |  | Mean, % | 95% CI | *P*-value† |  |
| Weekday  (Monday - Thursday) | 1.3 | 0.7, 2.0 | -22 | -55, 10 |  | 1.5 | 0.8, 2.6 | 2 | -34, 39 |  | -19 | -67, 29 | 0.435 |  |
| Weekend day  (Friday - Sunday) | 3.1 | 2.1, 4.7 | 2.6 | 1.5, 3.6 |  | 3.5 | 2.4, 4.4 | 3.6 | 2.4, 4.4 |  | -39 | -65, -13 | **0.003** |  |

s/d, servings per day (servings defined as serving sizes of 450 kJ or 250 ml discretionary drinks).

* Log-transformed mixed models estimating mean difference from baseline to follow-up. Adjusted for parental education and misreporting as fixed effects, and school and child as random effect.

† Log-transformed mixed models estimating mean difference between groups over time, time X group. Adjusted for parental education and misreporting as fixed effects, and school and child as random effect.
